# Histopathologic evaluation of the inflammatory factors and stromal cells in the endometriosis lesions: A case-control study

**DOI:** 10.18502/ijrm.v20i10.12266

**Published:** 2022-11-02

**Authors:** Ali Mohebbi, Vida Hojati, Masoumeh Majidi Zolbin, Reza Aflatoonian

**Affiliations:** ^1^Department of Biology, Damghan Branch, Islamic Azad University, Damghan, Iran.; ^2^Pediatric Urology and Regenerative Medicine Research Center, Gene, Cell, and Tissue Research Institute, Children's Medical Center, Tehran University of Medical Sciences, Tehran, Iran.; ^3^Department of Endocrinology and Female Infertility at Reproductive Biomedicine Research Center, Royan Institute for Reproductive Biomedicine, ACECR, Tehran, Iran.

**Keywords:** Endometriosis, PCNA, Stem cell, Inflammation.

## Abstract

**Background:**

Endometriosis is a multifaceted gynecological disorder defined as a benign estrogen-dependent chronic inflammatory process in which endometrial glands and stroma-like tissues are located outside the uterine cavity. It affects around 2-10% of all women during their reproductive years.

**Objective:**

This study aimed to evaluate the traffic of mesenchymal stem cells and inflammatory factors toward the lesions.

**Materials and Methods:**

Ten samples of normal endometrium and eutopic endometrium were studied as a control group and 10 ectopic samples were considered as a case group. Hematoxylin and eosin staining was used to evaluate stromal cells and inflammatory cells. Immunohistochemical staining was performed to show the presence of proliferating cell nuclear antigen in the lesions. The cells were digested and cultured in the laboratory to study cell proliferation. The number of cells and vessels were counted with Image J software, and data analysis was performed with Prism software.

**Results:**

Data analysis showed that the number of stromal cells and vessels in ectopic tissue were significantly higher than the control group (p < 0.001). Also, the number of inflammatory cells, including neutrophils, monocytes, lymphocytes, and macrophages, in the ectopic group was much higher than in the control group (p < 0.005).

**Conclusion:**

By expanding the number of blood vessels, blood flow increases, and cell migration to tissues is facilitated. The accumulation of inflammatory cells, especially macrophages, stimulates the growth of stem cells and helps implant cells by creating an inflammatory process.

## 1. Introduction

Endometriosis is a benign disease of the female reproductive tract defined by the presence of endometrial glands and stroma-like structures outside the uterine cavity (1, 2). 2-10% within the general female population and up to 50% with infertility were found to have endometriosis (3). This disease may be a major issue in female health, which causes a decreased quality of life (4, 5). Endometriosis's etiology and pathophysiology remain unknown. The classic and broadly acknowledged speculation for endometriosis, first proposed by Sampson in 1927, was the retrograde hypothesis. It was stated that the menstrual tissue contains endometrial glands and stroma appearing from the fallopian tubes reaching the peritoneal cavity through retrograde menstruation. It is implanting in areas such as fallopian tubes and various parts of the pelvic cavity (2). The lesions found exterior to the pelvis, such as the peripheral nervous system superior organs in the abdomen which are surrounded by viscera as well as border organs between thorax and abdomen wall cannot be justified by retrograde bleeding (6). As a result, additional mechanisms must contribute to endometriosis development. Meyer's theory of cellular metaplasia assumes that visceral and parietal peritoneal cells undergo metaplastic transformation into endometriosis lesions. Induction theory states that an endogenous (unspecified) biochemical agent can stimulate undifferentiated peritoneal cells to become endometrial tissue (7).

Adult stem cells are undifferentiated cells while maintaining their regenerative capacity is defined by their ability to produce different types of tissues distinct from the originated tissue (8). The endometrium contains stem/progenitor cells that play an important role in endometrial physiology, regeneration, repair, and endometriosis prognosis (9, 10). The recent focus of most studies was that endometriosis might originate from retrograde menstruation or the hematogenous/lymphatic spread of this stem cell population, and the source of endometriosis lesions may be stem cells that are circulating during menstruation (11, 12). Proliferating cell nuclear antigen (PCNA) is a factor for cell propagation expressing in the cell nucleus during the DNA synthesis (13). PCNA is a DNA polymerase δ side protein that takes part in amplifying, replicating, and repairing DNA strands, chromatin structure maintenance, chromosome segregation, and cell cycle progression and can be considered as a particular marker for the S stage of the cell cycle. Its expression level can reflect the grade of cell proliferation, so it can be considered as a common factor for cell proliferation activity (14).

**Figure 1:**
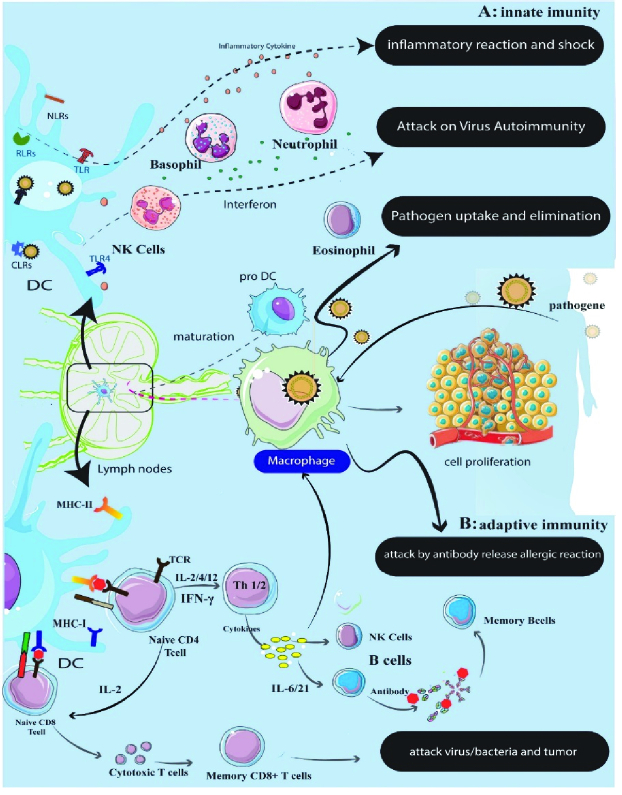
Immunity against invading agents is the responsibility of the innate immune system and the adaptive immune system. Macrophages do not act as scavengers to remove the ectopic cells of endometriosis. But, activated peritoneal macrophages and monocytes in the circulation of affected women can suppress the disease by secreting growth factors and cytokines that stimulate the proliferation of ectopic endometrium and inhibit the scavenger function of macrophages. Speed up cytokines and growth factors accelerate the implantation and growth of the ectopic endometrium by facilitating the attachment of ectopic tissue to the surface of the peritoneum and stimulating proliferation and angiogenesis. Various studies show a decrease in the activity of cytotoxic T cells, NK cells, cytokine secretion by T helper cells, and the production of autoantibody compounds by B lymphocytes in women with endometriosis.

In the last few decades, many studies have shown the role of immune system imbalances and immune responses in the etiology and pathophysiology of endometriosis (15, 16). In this regard, changes in the innate and acquired immune system (Figure 1) in women with endometriosis have been extensively studied. Even though there are 3 main hypotheses, the question arises as to why the disease occurs in a number of women while the reverse menstrual cycle occurs in many women. What explains the very different manifestations of this disease? Why does it occur severely in some women but not in all? Why is there a weak link between the disease and the severity of the symptoms?

Due to the unknown pathophysiology of endometriosis, we decided to investigate the histopathology of endometriosis and the most prevalent type of cells appearing in the ectopic lesions in this study.

## 2. Materials and Methods

### Study design and tissue collection

Thirty-two women with endometriosis and 20 women in the control group were included in this case-control study in Nikan hospital, Tehran, Iran during 2021. Finally, after applying the exclusion criteria, 10 women with endometriosis were studied as a case group, and 10 women, including 5 women in the secretory phase and 5 women in the proliferative phase as a control group. The mean age of the women in the case group was 32.5 and in the control group was 35.5.

Inclusion criteria for both case and control groups were as follows: all women at reproductive age who were candidate for laparoscopic surgery or laparotomy and willing to participate in the study. Moreover, the abdominal and pelvic pain in the case group was suspicious of endometriosis on physical examination and ultrasound findings. Infection or any inflammatory process in the abdominal cavity, hormone intake, and the need for sampling by pipele vaginal for control and eutopic tissue samples of virgin women in both groups were considered as an exclusion criteria. Women with endometriosis after biopsy of ectopic endometriosis tissue and pathological examination of women whose endometriosis was not confirmed were excluded from the study. Ten samples of normal endometrium and eutopic endometrium were studied as a control group and 10 ectopic samples were studied as a case group.

Samples were collected through pipele for the control group from the endometrium of 5 women in the secretory phase of menstruation and 5 women in the proliferative phase of the menstrual cycle who underwent surgery for reasons other than endometriosis. Samples of the case group were obtained from 10 women with stage 3 and 4 endometriosis. Eutopic endometrial specimens were sampled by vaginal pipele, and ectopic tissue was sampled by laparoscopy. The obtained samples were immediately transferred to the laboratory. After several steps of washing out with normal saline to clear the blood, 10 samples were transferred to the culture room immediately after sampling and cell culture was performed. Samples were transferred to formalin (Sigma-Aldrich, histological tissue fixative, USA, Product No. HT501128) for histopathological examination. The data were evaluated with immunohistochemistry and histopathologic tests.

### Hematoxylin and eosin staining

Sample from each group were transferred to formalin to prepare paraffin blocks. After preparing the paraffin embedded sample blocks, the blocks were stained with hematoxylin and eosin (Hematoxylin & Eosin, Merk, Germany, Product No. H3136). In the initial examination of the tissues of the control group in 2 phases of secretion and proliferation, as well as eutopic and ectopic tissues of the case with endometriosis, the presence of glands and stroma was evaluated histological tissue fixative to confirm the type of endometrial tissue. The number of stromal cells, including fibroblasts, mesenchymal cells, and hemorrhagic cells in the tissues, were examined. Also, the number of blood vessels in all tissues and ectopic tissue were examined. Finally, inflammatory cells, including neutrophils, macrophages, lymphocytes, and monocytes, were counted.

### Immunohistochemical staining of tissue sections 

To confirm the presence of the PCNA ${}^{+}$ (anti-PCNA Antibody (F-2): sc-25280, USA) population in the lesions, tissues were fixed in the paraformaldehyde (Thermo Fisher AAJ19943K2, USA) 4% and paraffin sections were deparaffinized and for 1 hr in temperature of 60 C. Following 3 passages in xylenes for 10 min, subsequently the sections were transferred to graded ethanol and finally washed in PBS. Antigen retrieval conducted for 10 min in sodium citrate. Internal peroxidase activity was blocked with 3% H ${}_{2}$ O ${}_{2}$ . Blocking performed with 10% rabbit serum and then primary and secondary PCNA antibody staining followed by hematoxylin counterstained. Hundred cells were counted at x10 magnification, and the percentage of the number of nuclei with positive PCNA was calculated.

### Cell culture

The endometrial cells were cultured after confirming the tissue type, so their images were evaluated under a microscope. The tissue was washed with PBS solution, and after homogenizing, the tissue was used for cell culture. A small portion of the endometrium or endometriosis lesion, about 0.5 × 0.5 cm, was transferred under the laminar hood. Because the sample obtained from the endometrium by biopsy with a pipelle was contaminated with a large number of red blood cells (RBC), the tissue was first placed in 1 cc of RBC lysis buffer solution (Roche, Germany Product No. 11814389001), and then the tissue was rewashed. The homogenized tissues were placed in a 1 cc collagenase solution at 0.1 mg/ml concentration. First, the falcon was shaken for 10 min and then placed in an incubator at 37 for 20 min. The samples were then stirred again for 15 min. The sample was then passed through a 70 m mesh filter placed over a 50 mL Falcon to separate debris from the cell solution. The tubes were centrifuged at 3000 g for 4 min at 4 C.

The supernatant was aspirated, and the cell plate was reconstituted in the tube with 1% PBS to ensure collagenase neutralization, and then centrifuged again. The supernatant was aspirated, and the cell plate was prepared for transfer to the culture medium. Finally, the cell pellet was vortexed with 2 ccs of culture medium containing Dulbecco's Modified Eagle Medium (Gibco, UK, Product No. 32500-035), Fetal Bovine Serum (Gibco, UK, Product No. 10270-106) 10%, and the antibiotics (penicillin and streptomycin), then incubated 200,000 cells in each 24 well plate at 37 C, humidity 70%, and CO ${}_{2}$ = 5% were transferred. To evaluate and compare the changes in the endometrial tissue of women with endometriosis. Cells were evaluated under a microscope following the culturing of cells derived from normal endometrial tissue and eutopic and ectopic endometrium.

### Ethical considerations

This study was conducted in the growth and development research center and approved by the Research Deputy and the Ethics Committee of Tehran University of Medical Sciences, Tehran, Iran on 13.07.2019 (Code: IR.TUMS.VCR.REC.1398.406) and conducted by the ethical standards according to the 1964 Declaration of Helsinki and all subsequent revisions. The informed consent form were obtained from all participants following the explanation about the goal of the study.

### Statistical analysis

After imaging 10 sections of each slide, the number of stromal cells, blood vessels, PCNA positive cells, and inflammatory cells were analyzed with Image J software (Digital Image Processing for Medical Applications, Cambridge University Press, ISBN 978-0-521-86085-7). Data were analyzed using Prism software. Student's *t* test, Mann–Whitney U test, and Chi-square test were applied for data analysis. A p-value ≤ 0.05 was considered statistically significant.

## 3. Results

The average weight was 68.1 kg in the case group and 68.5 kg in the control group. The height of the woman was 160.6-166 cm. The BMI in the case group was 24.9 and in the control group was 26.5 kg/m^2^. Two women in the case group had a familiar background of endometriosis, and the control group did not have any family background.

### Stromal cells, inflammatory cells, and blood vessels 

**Figure 2:**
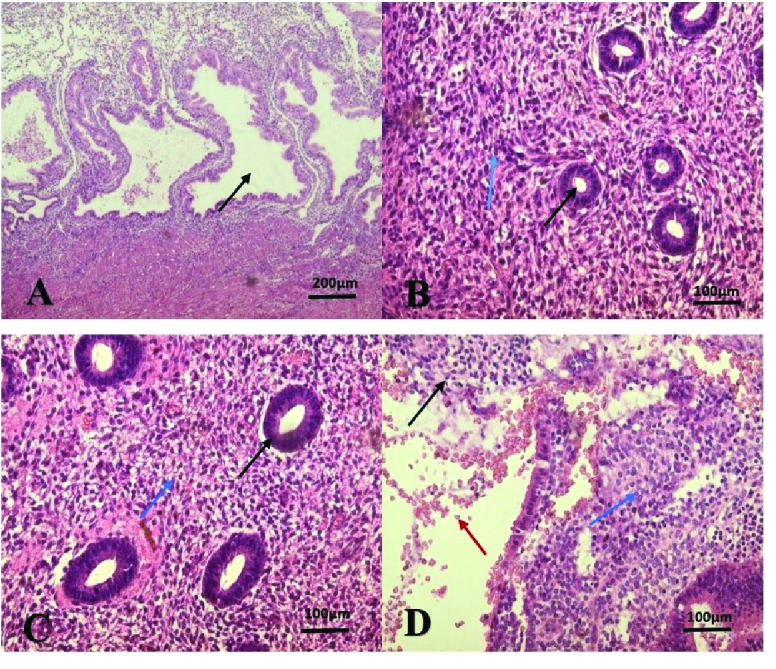
A: Microscopic view of the uterine endometrium in the control group, secretory phase, stromal cells, and large and twisted uterine glands (black arrow) (hematoxylin and eosin staining (H&E), 10 × magnification). B: Microscopic view of the uterine endometrium in the control group, proliferation phase, normal stromal cells (blue arrow), and uterine glands (black arrow) e (H&E staining, 20 × magnification). C: Microscopic view of the uterine endometrium in eutopic group, stromal cells (blue arrow) and uterine glands (black arrow) almost normal (H&E staining, 20 × magnification). D: Microscopic view of the endometrium in the Ectopic group, the proliferation of endometrial cells, abundant stromal cells that form the glands, hemorrhage with stromal cells, and a very large number of mesenchymal cells. Stromal cells in this image include fibroblasts with a bolder oval nucleus (black arrow), a large number of mesenchymal cells with a round or large oval nucleus (blue arrow), and hemorrhage (red arrow).

**Figure 3:**
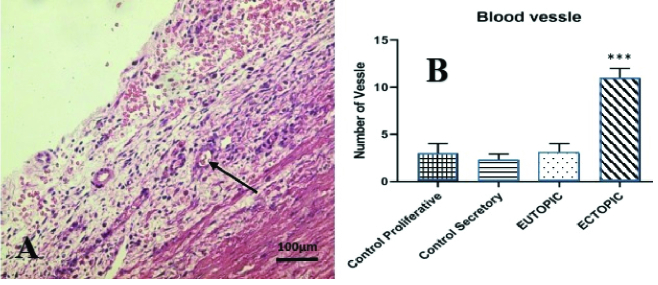
A: Microscopic view of the endometrium in the ectopic group, with abundant blood vessels (arrow) and bleeding. B: Significant difference in the number of vessels (10 regions per sample) in the ectopic group with other groups with has been demonstrated with ${}^{***}$ PV < 0.001.

**Figure 4:**
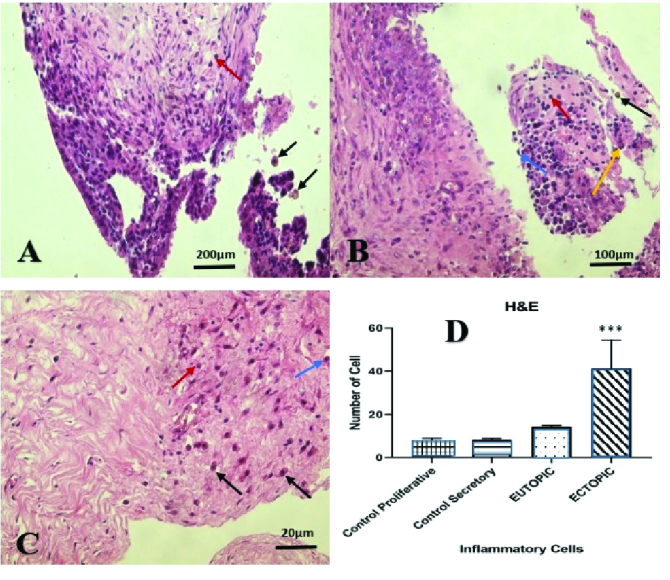
A: Microscopic view of the endometrium in the Ectopic group, with abundant inflammatory cells (black arrow, macrophage) (red arrow, lymphocyte = round nucleus) (H&E staining, 10X magnification). B: Endometrial microscopic view in the ectopic group, with abundant inflammatory cells (black arrow, macrophage) (red arrow, lymphocyte = round nucleus) (blue arrow, monocyte = bean nucleus) (yellow arrow, neutrophil = multinucleated nucleus) (color hematoxylin and eosin combination, 20X magnification). C: Endometrial microscopic view in the ectopic group, abundant inflammatory cells with fibrosis (black arrow, macrophage) (red arrow, lymphocyte = round nucleus) (blue arrow, monocyte = bean nucleus) (H&E staining, 40X magnification). D: Significant difference in the number of inflammatory cells (neutrophils-lymphocytes-macrophages- plasmocytes) between the ectopic group with control and Eutopic with p < 0.005, and are indicated with ${}^{***}$ .

The histological evaluation of ectopic tissue demonstrated presence of stromal cells, including fibroblast and mesenchymal cells, of which the larger portion belongs to MSCs (Figure 2). The Image J analysis represents the higher incidence of these cells compared to eutopic and control endometrium tissues (p < 0.0005). The number of stromal cells in the eutopic group also showed a significant difference compared to both control groups in the proliferative and secretory phases (p < 0.005) (Table I). As illustrated in figure 3, the ectopic group had more blood vessels than the other groups (p < 0.0001). In the H&E study, the total number of inflammatory cells, including neutrophils, lymphocytes, macrophages, and plasmocytes were considerably greater in ectopic endometrial tissue than in the control group during secretory and proliferative phases, and in the eutopic endometrial group (p < 0.005) (Table I). Fibrous cells were seen in a higher magnification of ectopic tissue along with these inflammatory cells (Figure 4).

### Cells culture

**Figure 5:**
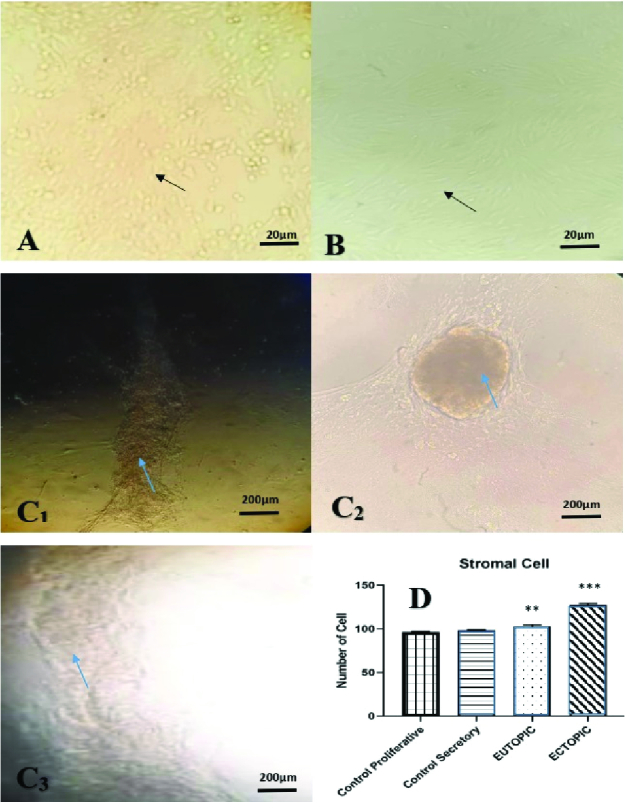
A: View of normal endometrial cell culture magnification 10X. B: Eutopic endometrial tissue cell culture with 10X magnification. C: Ectopic endometrial tissue cell culture view (arrows mesenchymal stem cell colonies). D: Significant difference in the number of stromal cells between the ectopic group with control and eutopic with p < 0.0005 and between the eutopic and control with p < 0.005. According to figure 1, which is represented respectively with (**) and (***).

Following cell culture, the endometrial tissue cells grew as spherical cells. However, in the cells of endometriosis ectopic tissues, due to many stem cells next to the fibroblast cells of the endometrium, golden-colored colonies of stem cells were observed. These colonies were not seen as eutopic and controlled endometrial groups (Figure 5).

### PCNA

**Figure 6:**
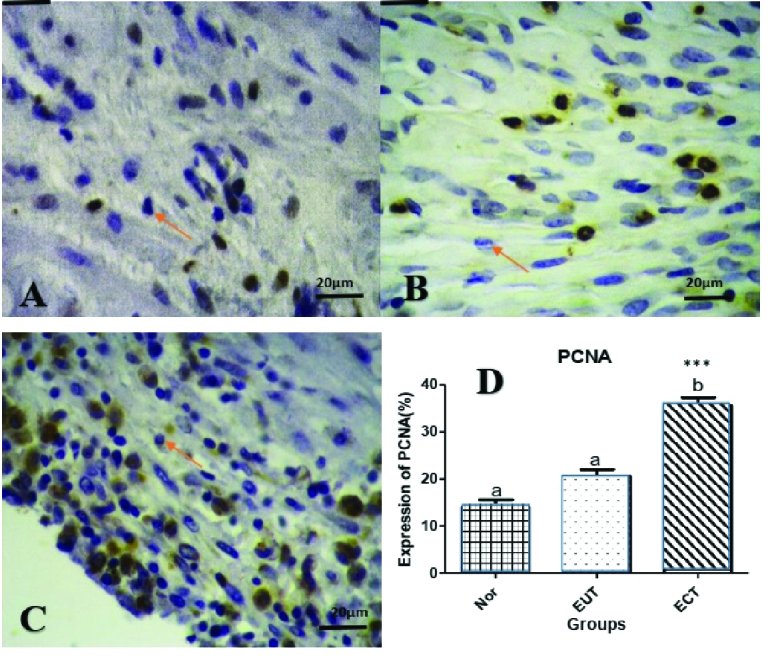
Microscopic view of PCNA antibody expression in the endometrium (arrow) A: Normal, B: Eutopic, C: Ectopic endometrium in the proliferative phase, D: Endometrial cell proliferation, in Ectopic tissue compared with Eutopic tissue and control (p < 0.0002) and presented with ${}^{***}$ .

By studying immunohistochemistry on ectopic, eutopic, and normal endometrial tissues in the proliferative phase using PCNA antibody to determine the rate of cell proliferation in each group, the number of cells expressing this antibody was counted using Image J software and shown in table I. Data analysis with Prism software showed that antibody expression was higher in the ectopic group with a higher level than in the 2 eutopian and the control groups (p < 0.01) (Figure 6).

**Table 1:** Mean of differentiated stromal cells, PCNA, number of vessels, and inflammatory cells in 10 sections of each slide


**Sample type**	**Mean of stromal cells**	**Mean of PCNA**	**Mean of inflammatory cells**
**Control (secretory phase)**	97	- 8
**Control (proliferative phase)**	98	14.07	9
**Eutopic**	105	20.57	14
**Ectopic**	129	35.63	49
**Tukey's multiple comparisons tests**	Mean diff.	Adjusted p-value
**Control proliferative vs. control secretory**	-2.000	0.20
**Control proliferative vs. eutopic**	-6.667	0.001
**Control proliferative vs. ectopic**	-31.33	< 0.001
**Control secretory vs. eutopic**	-4.667	0.001
**Control secretory vs. ectopic**	-29.33	< 0.001
**Eutopic vs. ectopic**	-24.67	< 0.001
**Tukey's multiple comparison test**	Mean diff.	Adjusted p-value
**Control proliferative vs. eutopic**	-6.500	> 0.05
**Control proliferative vs. ectopic**	-21.57	0.001
**Eutopic vs. ectopic**	-15.07	0.001
**Tukey's multiple comparisons test**	Mean diff.	Adjusted p-value
**Control proliferative vs. control secretory**	0.6667	0.80
**Control proliferative vs. eutopic**	0.000	> 0.99
**Control proliferative vs. ectopic**	-8.000	< 0.001
**Control secretory vs. eutopic**	-0.6667	0.80
**Control secretory vs. ectopic**	-8.667	< 0.001
**Eutopic vs. ectopic**	-8.000	< 0.001
**Tukey's multiple comparisons test**	Mean diff.	Adjusted p-value
**Control proliferative vs. control secretory**	-0.3333	> 0.99
**Control proliferative vs. eutopic**	-6.333	0.66
**Control proliferative vs. ectopic**	-33.33	0.001
**Control secretory vs. eutopic**	-6.000	0.69
**Control secretory vs. ectopic**	-33.00	0.001
**Eutopic vs. ectopic**	-27.00	0.04
After imaging 10 sections of each slide, the number of stromal cells, blood vessels, PCNA positive cells, and inflammatory cells were analyzed with Image J software (Digital Image Processing for Medical Applications. Cambridge University Press. ISBN 978-0-521-86085-7). All the information was analyzed using Prism. Student's *t* test, Mann–Whitney test, and Chi-square test were applied to data analysis. A p-value ≤ 0.05 was considered statistically significant, PCNA: Proliferating cell nuclear antigen			

## 4. Discussion

The present study represented that the number of stromal cells, especially mesenchymal stem cells in ectopic tissue, is more numerous than endometrial tissue in both control and eutopic tissue. This may indicate that these cells are composed of endometrial stem cell aggregation (17, 18). This can be accompanied by an increase in the expression of genes involved in the function of stem cells (19). Still, some recent studies have shown the presence of bone marrow-derived mesenchymal stem cells in ectopic tissues, which may indicate that the ectopic endometrium can recruit stem cells from different parts of the body (20, 21). On the other hand, the observation of many blood vessels in ectopic tissue in this study indicates the blood supply needed for migration, transplantation, replacement, and growth of stem cells. The expression of vascular endothelial growth factor, a key mediator of local angiogenesis that stimulates the proliferation of vascular endothelial cells in ectopic tissue, was considerably higher (22, 23). This study showed that an increase in the expression of PCNA in the presence of a large number of stromal cells, especially mesenchymal stem cells in ectopic tissue, increases the rate and rate of cell growth. Ectopic cells culture in the in vitro environment and high growth rate, caused golden-colored colonies of mesenchymal stem cells.

Our study also showed that the number of inflammatory cells, including lymphocytes, monocytes, neutrophils, and especially macrophages, within ectopic tissue increased dramatically. Increasing the number of inflammatory cells in ectopic tissue and peritoneal fluid can stimulate the growth of endometriosis. Laboratory studies have shown that peritoneal macrophages in women with endometriosis express cytokines IL-6, IL-1B, and tumor necrosis factor more than in women with benign abnormalities (24). Kempuraj and colleagues showed that the number of highly active mast cells increased in peritoneal endometrial lesions compared to the eutopic endometrium (25). Failure in immune response at the cellular level will result in impaired removal of endometrial cells from peritoneal cavity and ectopic cell. So far it has been confirmed that the activity status of the cytotoxic T cells, NK cells, cytokine secretion by helper T cells, and production of autoantibodies by B lymphocytes will be reduced in patients with endometriosis and it will increase the inflammation and illness, especially with severe pain during menstrual bleeding (26, 27). The use of nonsteroidal anti-inflammatory medicines frequently alleviates pain and other symptoms associated with inflammatory indicators (28, 29).

Some studies have shown that PCNA is associated with tumor differentiation, penetration, recurrence and lymph node, or organ metastasis (30, 31). PCNA expression levels are known to be elevated in endometrial hyperplasia and endometriosis. Decreased size of endometriosis lesions is associated with decreased PCNA levels, and PCNA levels are directly related to the size of endometriosis lesions (32).

It may be possible to search for the missing link in the etiology of endometriosis in the basal immunity of endometriosis, which plays an important role in developing and growing the disease (33-35). Although ectopic endometrial cells are recognized by the cells of the peritoneal cavity immune system, they are, as a rule, not devastated and elude the immune system. Therefore, the weakening or modulation of the local immune system in the peritoneal cavity is one of the main factors in the growth of endometrial parts in the peritoneal cavity of women with endometriosis. The susceptibility of implantation and growth of endometrial cells may be due to the lack of regulation of the immune system clearance mechanism (36, 37).

Macrophages constitute the majority of peritoneal leukocytes, and based on existing research, they constitute about 90% of peritoneal cells (38). Macrophages do not act as scavengers to remove the abnormal endometriosis cells, rather it activates the peritoneal macrophages and circulating monocytes in endometriosis women by secreting growth factors and cytokines that stimulate ectopic endometrial proliferation and inhibit our scavenger function (39, 40).

## 5. Conclusion

Endometriosis is a common disease in women at fertility age as a social and welfare problem. Classical theories in the pathophysiology of this disease alone cannot justify all types and variations in disease severity. Our histopathological examination showed that stem cells in ectopic tissue and high expression of PCNA increases the growth rate of this tissue. On the other hand, increased angiogenesis in ectopic tissue, and increasing blood flow to these tissues, allow stem cells to migrate from other parts of the body to this site.

The presence of many inflammatory cells can also stimulate the angiogenic factors in these tissues. Identification of a large number of these inflammatory cells, especially macrophages in ectopic tissue, shows that these cells not only perform their main function as xenophagous and destroy foreign cells but also themselves as a stimulus for the growth of these cells. Increased inflammation can facilitate the implantation of migrating cells and retrograde blood cells, so it is necessary to characterize the type of these cells to design the therapeutic targets. Identifying and blocking these immune factors with a pharmaceutical approach can shed light on future potential treatments.

##  Conflict of Interest 

The authors declare that they have no competing interest.
